# Genetic dissection of bull fertility in US Jersey dairy cattle

**DOI:** 10.1111/age.12710

**Published:** 2018-08-14

**Authors:** F. M. Rezende, G. O. Dietsch, F. Peñagaricano

**Affiliations:** ^1^ Department of Animal Sciences University of Florida Gainesville FL 32611 USA; ^2^ Faculdade de Medicina Veterinária Universidade Federal de Uberlândia Uberlândia MG 38400‐902 Brazil; ^3^ University of Florida Genetics Institute University of Florida Gainesville FL 32610 USA

**Keywords:** genomic scan, non‐additive effects, pathway analysis, sire conception rate

## Abstract

The service sire has been recognized as an important factor affecting herd fertility in dairy cattle. Recent studies suggest that genetic factors explain part of the difference in fertility among Holstein sires. The main objective of this study was to dissect the genetic architecture of sire fertility in US Jersey cattle. The dataset included 1.5 K Jersey bulls with sire conception rate (SCR) records and 96 K single nucleotide polymorphism (SNP) markers spanning the whole genome. The analysis included whole‐genome scans for both additive and non‐additive effects and subsequent functional enrichment analyses using KEGG Pathway, Gene Ontology (GO) and Medical Subject Headings (MeSH) databases. Ten genomic regions located on eight different chromosomes explained more than 0.5% of the additive genetic variance for SCR. These regions harbor genes, such as *PKDREJ*,*EPB41L2*,*PDGFD*,*STX2*,*SLC25A20* and *IP6K1*, that are directly implicated in testis development and spermatogenesis, sperm motility and the acrosome reaction. In addition, the genomic scan for non‐additive effects identified two regions on BTA11 and BTA25 with marked recessive effects. These regions harbor three genes—*FER1L5*,*CNNM4* and *DNAH3*—with known roles in sperm biology. Moreover, the gene‐set analysis revealed terms associated with calcium regulation and signaling, membrane fusion, sperm cell energy metabolism, GTPase activity and MAPK signaling. These gene sets are directly implicated in sperm physiology and male fertility. Overall, this integrative genomic study unravels genetic variants and pathways affecting Jersey bull fertility. These findings may contribute to the development of novel genomic strategies for improving sire fertility in Jersey cattle.

## Introduction

Reproductive management of dairy cows has advanced significantly in the past 20 years with the advent of ovulation synchronization protocols, improvements in nutrition and cow comfort and, more recently, the incorporation of fertility and longevity traits into genetic selection programs. However, despite these advances, reproductive efficiency of dairy cattle remains suboptimal, resulting in significant economic losses for the dairy industry (Inchaisri *et al*. 2010). Bull infertility is often overlooked as a potential cause of reproductive inefficiency. Nonetheless, some studies have revealed that a significant percentage of reproductive failure in dairy cattle is attributable to service sire subfertility (DeJarnette *et al*. 2004; Amann & DeJarnette 2012). Indeed, the fertility of the bull is critical in determining herd reproductive performance, and hence, it should not be ignored in dairy cattle breeding schemes aimed at improving reproductive efficiency (Amann *et al*. 2018; Taylor *et al*. 2018).

Dairy bull fertility has been traditionally evaluated in the laboratory using different semen production and quality attributes such as sperm morphology, sperm concentration and sperm motility (DeJarnette *et al*. 2004). Unfortunately, these sperm quality traits explain only part of the differences observed in fertility among dairy sires (Parkinson 2004). Alternatively, bull fertility can be evaluated directly using conception rate records. In this sense, since 2008, the US dairy industry has had access to a phenotypic evaluation of male fertility called sire conception rate (SCR) (Kuhn & Hutchison 2008). This bull fertility evaluation is based on a large, nationwide database of confirmed pregnancy records. Interestingly, there is a remarkable variation in SCR among dairy sires—more than a 10% conception rate difference between high‐fertility and low‐fertility bulls (Peñagaricano *et al*. 2012).

Our group has been investigating potential genetic factors underlying the observed variation in SCR in dairy cattle. We have identified regions on BTA21 and BTA25 that explain a significant amount of additive genetic variance (Han & Peñagaricano 2016). In addition, we recently reported significant non‐additive effects on BTA8, BTA9, BTA13 and BTA17 (Nicolini *et al*. 2018). Notably, all these genomic regions harbor genes with known roles in sperm physiology and male biology. It should be noted that all these studies were performed on Holstein cattle. Little is known, however, about the genomic architecture underlying SCR in Jersey cattle. The Jersey breed is the second most important breed in the US, representing at least 12% of the national cow population. The proportion of Jersey semen sold domestically by National Association of Animal Breeders’ members increased from 6% in 2000 to 13% in 2016 (Dechow *et al*. 2018). Although the Jersey breed in general has a greater conception rate than does the Holstein breed, its reproductive performance remains subpar (Norman *et al*. 2009). Female fertility traits are routinely evaluated and included in US Jersey selection programs, while bull fertility has received scarce attention.

The objective of this study was to dissect the genomic architecture underlying SCR in US Jersey dairy cattle. Alternative genome‐wide mapping approaches and gene‐set analyses were performed to identify genomic regions, individual genes and genetic mechanisms affecting Jersey sire fertility. The identification of fertility genes and pathways would provide better understanding of this complex trait and may point out new strategies for improving Jersey bull fertility via marker‐assisted selection.

## Materials and methods

### Phenotypic and genotypic data

Sire conception rate, a phenotypic evaluation of service sire fertility, has been provided to the US dairy industry since August 2008, initially by the Animal Improvement Programs Laboratory of the US Department of Agriculture and now by the Council of Dairy Cattle Breeding (CDCB). This bull fertility evaluation is based on cow field data, considering both factors related to the sire under evaluation (e.g. age and AI stud) and factors associated with the cow that receives the unit of semen (e.g. herd‐year‐season, age, parity and milk yield) (Kuhn & Hutchison 2008; Kuhn *et al*. 2008). The SCR trait is defined as the expected difference in conception rate of a given bull compared to the mean of all other evaluated bulls. The SCR evaluation is intended as phenotypic rather than a genetic evaluation because the estimates include both genetic and non‐genetic effects.

The entire evaluation of US Jersey bull fertility was used in this study. Specifically, a total of 6353 SCR records were available from 1557 Jersey bulls. These records were obtained from 27 consecutive SCR evaluations between August 2008 and August 2017. The SCR records and their reliabilities, calculated as function of the number of breedings, are freely available at the CDCB website (https://queries.uscdcb.com/eval/summary/scr_menu.cfm).

Genotype data for 107 371 single nucleotide polymorphism (SNP) markers were available for 1487 Jersey bulls with SCR evaluation. Genotype data were kindly provided by the Cooperative Dairy DNA Repository. The SNP markers that mapped to the sex chromosomes, were monomorphic or had minor allelic frequency less than 1% or calling rate less than 90% were removed from the dataset. After quality control, a total of 95 705 SNP markers were retained for subsequent genomic analysis.

### Genome‐wide mapping: additive effects

The relevance of additive effects was investigated using the single‐step genomic best linear unbiased prediction (ssGBLUP) method (Aguilar *et al*. 2010; Wang *et al*. 2012). The ssGBLUP combines all the available phenotypic, pedigree and genotypic information. This analysis was implemented within the framework of the classical repeatability animal model:

y=Xβ+Zu+Wpe+e,

where **y** is the vector of SCR records, ***β*** is the vector of fixed effects included in the model, **u** is the vector of the random animal effects, **pe** is the vector of random permanent environmental and non‐additive effects and **e** is the vector of random residual effects. The matrices **X**,** Z** and **W** are the incidence matrices relating phenotypic records to fixed, animal and permanent environment effects respectively. The random effects are assumed to follow a multivariate normal distribution,

$$\left(\begin{array}{c} u\\ \mathrm{pe}\\ e \end{array}|\sigma_{u}^{2},\sigma_{\mathrm{pe}}^{2},\sigma_{e}^{2}\right)∼N\left[0,\left(\begin{array}{ccc} H\sigma_{u}^{2} & 0 & 0\\ 0 & I\sigma_{\mathrm{pe}}^{2} & 0\\ 0 & 0 & R^{-1}\sigma_{e}^{2} \end{array}\right)\right],$$

where $\sigma_{u}^{2}$, $\sigma_{\mathrm{pe}}^{2}$ and $\sigma_{e}^{2}$ are the animal additive genetic, permanent environmental and residual variances respectively. The classical pedigree relationship matrix **A** is replaced by **H**, which combines pedigree and genotypic information (Aguilar *et al*. 2010). The combined pedigree–genomic relationship matrix **H** ^−1^ was calculated as follows:

$$H^{-1}=A^{-1}+\left[\begin{array}{cc} 0 & 0\\ 0 & G_{1}^{-1}+A_{22}^{-1} \end{array}\right],$$

where $G_{1}^{-1}$ is the inverse of the genomic relationship matrix and $A_{22}^{-1}$ is the inverse of the pedigree‐based relationship matrix of genotyped animals. In our case, **G**_1_ has the dimensions 1741 × 1741, and it was created using 1487 sires with both SCR records and genotype data plus 254 genotyped sires with no SCR records. The **A** matrix (5207 × 5207) was calculated based on a five‐generation pedigree downloaded from the CDCB website. The residual matrix **R** is a diagonal matrix with its elements representing the reliabilities of the SCR values.

Given the vector of genomic estimated breeding values (GEBV, **û**), the SNP effects can be estimated as $\hat{s}={\mathrm{DM}}^{\prime }{\left[{\mathrm{MDM}}^{\prime }\right]}^{-1}\hat{u}$, where **M** is a matrix relating genotypes of each locus and **D** is a diagonal matrix of weights of SNPs (Wang *et al*. 2012). Candidate regions associated with SCR were identified based on the amount of additive genetic variance explained by 1.5‐Mb windows of adjacent SNPs evaluated across the entire bovine genome. The percentage of additive genetic variance explained by a given SNP window was calculated as

$$\frac{\text{Var}(w_{i})}{\sigma_{u}{}^{2}}\times 100=\frac{\text{Var}(\sum_{j=1}^{B}M_{j}{\hat{s}}_{j})}{\sigma_{u}^{2}}\times 100,$$

where *w*_*i*_ is the genetic value of the *i*th genomic window under consideration, *B* is the total number of adjacent SNPs within the 1.5‐Mb window and ${\hat{s}}_{j}$ is the marker effect of the *j*th SNP within the *i*th window. All the ssGBLUP computations were performed using the BLUPF90 family of programs from Ignacy Misztal and collaborators, University of Georgia.

### Genome‐wide mapping: non‐additive effects

The relevance of non‐additive effects, namely dominance, recessive and overdominance effects, on SCR was evaluated on a genome‐wide scale using a two‐step mixed‐model‐based approach (Aulchenko *et al*. 2007a, 2010).

In the first step, the following mixed model was fitted: **y** = **X** ***β*** + **Zu** + **e**. Note that only one record per animal was considered, and hence, for bulls with multiple evaluations, the most reliable SCR record was used. The random effects were assumed multivariate normal with $u∼N(0,G_{2}\sigma_{u}^{2})$ and $e∼N(0,I\sigma_{e}^{2})$. The matrices **G**_2_ and **I** have dimensions 1487 × 1487, i.e. the total number of Jersey bulls with both phenotypic and genotypic data. The variance–covariance matrix of this model was estimated as $V_{0}={\mathrm{ZG}}_{2}Z^{\prime }\sigma_{u}^{2}+I\sigma_{e}^{2}$.

In the second step, the following model was fitted for every SNP: **y** = **X** ***β*** + *X*_SNP_ *β*_SNP_ + ***ϵ***, assuming $e∼N(0,V_{0}\sigma_{e}^{2})$. Every SNP genotype *X*_SNP_ (0, 1, 2) was recoded using single numeric variables as (0, 1, 1), (0, 0, 1) and (0, 1, 0) for testing potential dominance, recessive and overdominance effects respectively. The significance of each non‐additive effect was tested using the following test statistic:

$$z=\frac{X_{\mathrm{SNP}}^{\prime }V_{0}^{-1}\left(y-X\hat{\beta }\right)}{\sqrt{X_{\mathrm{SNP}}^{\prime }V_{0}^{-1}X_{\mathrm{SNP}}}},$$

which approximates the Wald test and is asymptotically standard normal. These analyses were performed using the R package mixabel (Aulchenko *et al*. 2007b). Genome‐wide results were corrected for possible inflation of the test statistics using the function VIFGC implemented in the R package genabel (Tsepilov *et al*. 2013).

### Gene‐set analysis

Following Peñagaricano *et al*. (2013), a three‐step gene‐set analysis was implemented.

#### Assignment of SNPs to genes

A given SNP marker was assigned to a particular gene if it was located within or at most 15‐kb either upstream or downstream of the gene. This was implemented using the Bioconductor R package biomart (Durinck *et al*. 2005) based on the information provided by the UMD3.1 bovine genome assembly (Zimin *et al*. 2009). The distance of 15 kb was used to capture proximal regulatory regions that may lie outside, but close to, the gene. Based on the results of the ssGBLUP, an arbitrary threshold of 1% of the SNP effects was used to define significant SNP markers; in this context, significant genes were defined as those genes that were flagged by at least one significant SNP.

#### Assignment of genes to gene sets

The KEGG Pathway (Ogata *et al*. 1999), Gene Ontology (GO) (Ashburner *et al*. 2000) and Medical Subject Headings (MeSH) (Nelson *et al*. 2004) databases were used to define gene sets. Genes assigned to the same functional term can be considered as members of a group of genes (aka gene set) that share some particular properties, typically their involvement in the same biological process or molecular function.

#### Pathway‐based association analysis

The association of a given gene set with SCR was assessed using a hypergeometric test, also known as Fisher's exact test. The *P‐*value of observing *g* significant genes in the term was calculated by

$$P\;\text{value}=1-\sum_{i=0}^{g-1}\frac{\left(\begin{array}{c} S\\ i \end{array}\right)\left(\begin{array}{c} N-S\\ k-i \end{array}\right)}{\left(\begin{array}{c} N\\ k \end{array}\right)},$$

where *S* is the total number of genes associated with SCR, *N* is the total number of genes analyzed in the study and *k* is the total number of genes in the gene set under consideration (Gambra *et al*. 2013; Abdalla *et al*. 2016). The KEGG and GO gene‐set enrichment analyses were performed using the R package goseq (using method hypergeometric) (Young *et al*. 2010), and the MeSH enrichment analysis was carried out using the R package meshr (Morota *et al*. 2015; Tsuyuzaki *et al*. 2015).

## Results

### Whole‐genome scan for additive effects

**Figure 1:**
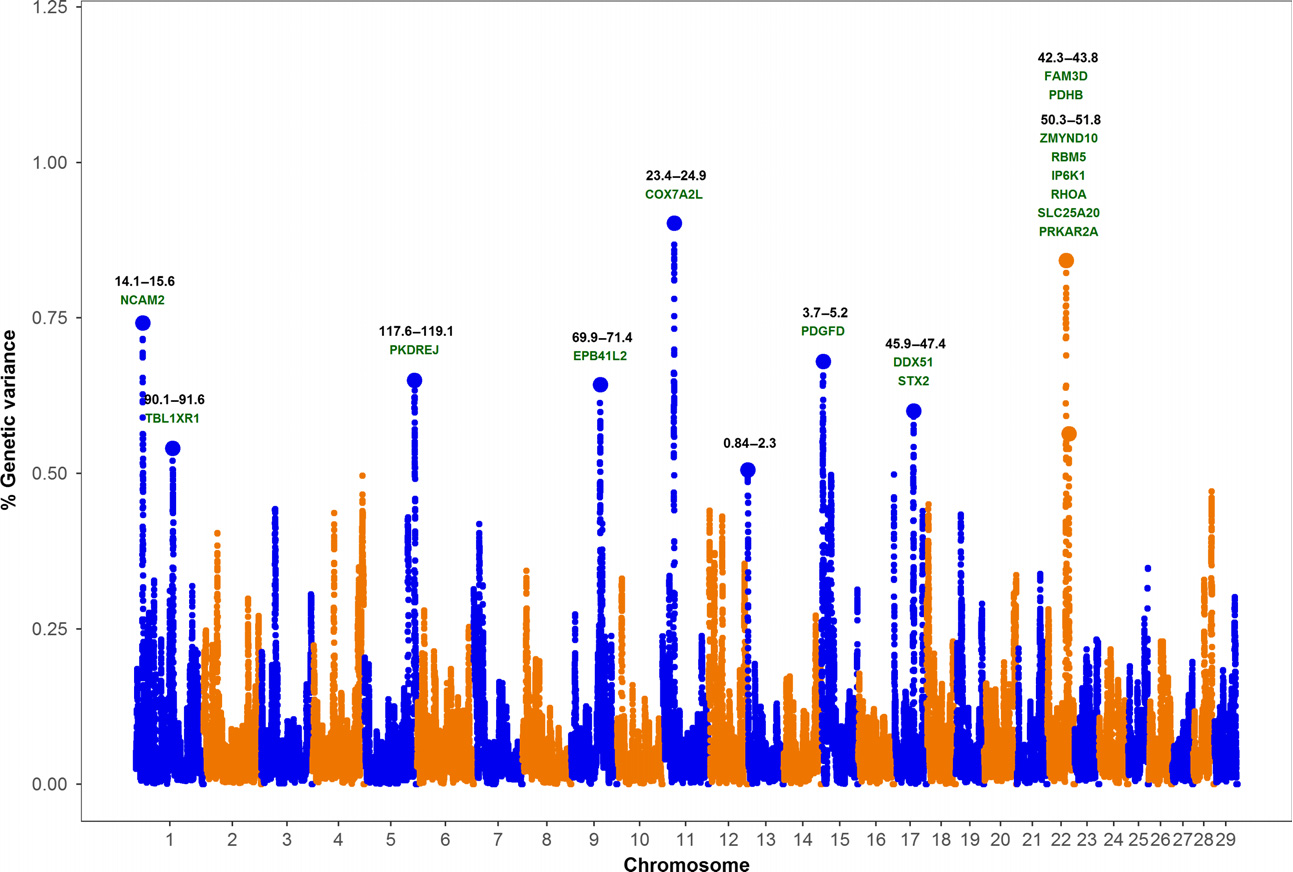
Whole‐genome scan for sire conception rate: percentage of additive genetic variance explained by 1.5 Mb SNP‐windows across the entire genome. The exact position of each SNP‐window (Mb) is highlighted in black, and putative genes affecting bull fertility are highlighted in green.

The importance of additive effects on SCR was evaluated using ssGBLUP. This method was originally developed for genomic prediction and later was extended for performing gene mapping. The proportion of the additive genetic variance explained by 1.5‐Mb SNP windows across the entire bovine genome is shown in Fig. 1. Ten different genomic regions, located on chromosomes BTA1, BTA5, BTA9, BTA11, BTA13, BTA15, BTA17 and BTA22, explain more than 0.5% of the additive genetic variance for SCR. The genomic region that explains the highest percentage of additive genetic variance (0.90%) was located on BTA11 (23.4–24.9 Mb). This region harbors *COX7A2L*, encoding one subunit of the cytochrome c oxidase that promotes respiratory supercomplex assembly and regulates energy generation, possibly involved in sperm motility. Two genomic regions on BTA22 (42.3–43.8 and 50.3–51.8 Mb) jointly explained 1.4% of the additive genetic variance. Remarkably, these two regions harbor several candidate genes for bull fertility, including *ZMYND10* and *SLC25A20*, which are directly involved in sperm motility; *IP6K1* and *RBM5*, which play critical roles in male germ cell maintenance and differentiation; and *PDHB,* which is implicated in sperm capacitation. Moreover, two 1.5‐Mb SNP windows located on BTA1 (14.1–15.6 and 90.1–91.6 Mb) explained a substantial amount of the genetic variance (1.3%); these regions harbor genes *NCAM2* and *TBL1XR1*, which are involved in testis development and spermatogenesis. In addition, three regions distributed on BTA5 (117.6–119.1 Mb), BTA9 (69.9–71.4 Mb) and BTA15 (3.7–5.2 Mb) explained roughly 0.65% of the additive genetic variance. At least three genes directly implicated in sperm physiology and male fertility, namely *PKDREJ*,* EPB41L2* and *PDGFD*, are located in these three regions respectively. Finally, the region identified on BTA17 (45.9–47.4 Mb) harbors two candidate genes: *DDX51*, which is involved in spermatogenesis and testis development, and *STX2*, which plays an active role in the acrosome reaction.

### Whole‐genome scan for non‐additive effects

**Figure 2:**
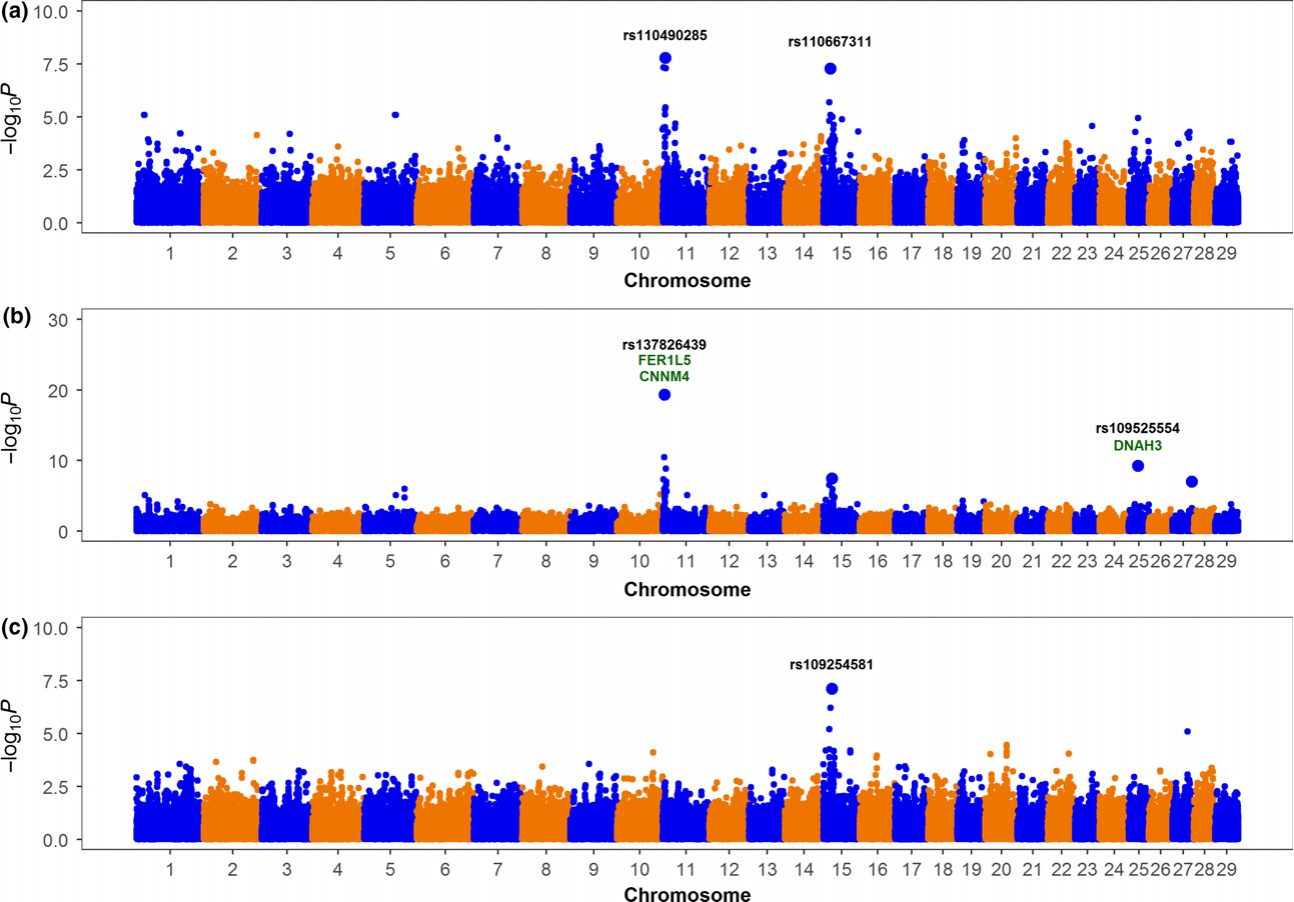
Whole‐genome scan for sire conception rate: significance of dominance (top), recessive (middle) and overdominance (bottom) effects across the entire genome. The SNP names are highlighted in black, and putative genes affecting bull fertility are highlighted in green.

The potential role of dominance, recessive and overdominance effects on Jersey sire fertility was evaluated using an efficient two‐step mixed‐model‐based approach. Three genomic regions, located on BTA11, BTA25 and BTA27, showed either marked recessive or dominance effects on SCR after Bonferroni correction (adjusted *P*‐value < 0.01) (Fig. 2). Full descriptions of the most significant SNP markers, including position, allele and genotypic frequencies and genetic effects, are displayed in Table 1. Note that the SNP markers on BTA11, namely rs42754787, rs137826439 and rs110490285, were highly correlated (high linkage disequilibrium), and hence, it is very likely that they represent the same genetic signal. Interestingly, the three regions on BTA11, BTA25 and BTA27 showed negligible additive effects, and hence, we can conclude that the mode of gene action of these loci is purely non‐additive. No region showed pure overdominance effects.

**Table 1:** Most significant non‐additive SNP loci associated with sire conception rate in US Jersey cattle

Marker	Chromosome	Position	MAF	Frequency	$\hat{\beta }\pm \text{SE}$	*P*_NonAdd_	*P*_Add_
Dominance							
rs110490285	11	4 009 903	0.13	25 (AA)	2.57 ± 0.45	1.68 × 10^−8^	5.97 × 10^−3^
				338 (AB)			
				1124 (BB)			
rs110667311	15	17 426 575	0.11	1150 (AA)	−0.88 ± 0.16	5.34 × 10^−8^	1.71 × 10^−8^
				333 (AB)			
				4 (BB)			
Recessive							
rs42754787	11	977 617	0.07	1292 (AA)	−4.55 ± 0.83	4.79 × 10^−8^	2.72 × 10^−4^
				180 (AB)			
				7 (BB)			
rs137826439	11	2 643 308	0.09	1211 (AA)	−6.34 ± 0.69	4.80 × 10^−20^	1.73 × 10^−4^
				240 (AB)			
				10 (BB)			
rs109525554	25	19 173 921	0.14	1082 (AA)	−5.57 ± 0.89	6.05 × 10^−10^	4.86 × 10^−3^
				399 (AB)			
				6 (BB)			
rs41649133	27	42 442 485	0.05	1327 (AA)	−8.29 ± 1.55	1.01 × 10^−7^	9.68 × 10^−1^
				158 (AB)			
				2 (BB)			
Overdominance							
rs109254581	15	21 106 423	0.11	5 (AA)	−0.93 ± 0.16	7.75 × 10^−8^	5.35 × 10^−8^
				331 (AB)			
				1151 (BB)			

MAF, minor allele frequency; *P*_Add_, *P*‐value additive model; *P*_NonAdd_, *P*‐value non‐additive model.

The significant region detected on BTA11 harbors at least two putative candidate genes for service sire fertility, namely *FER1L5* and *CNNM4*. *FER1L5* is directly involved in spermatogenesis, and *CNNM4* is implicated in sperm capacitation and sperm motility. Moreover, the significant region on BTA25 harbors the gene *DNAH3*, which also affects sperm motility.

**Figure 3:**
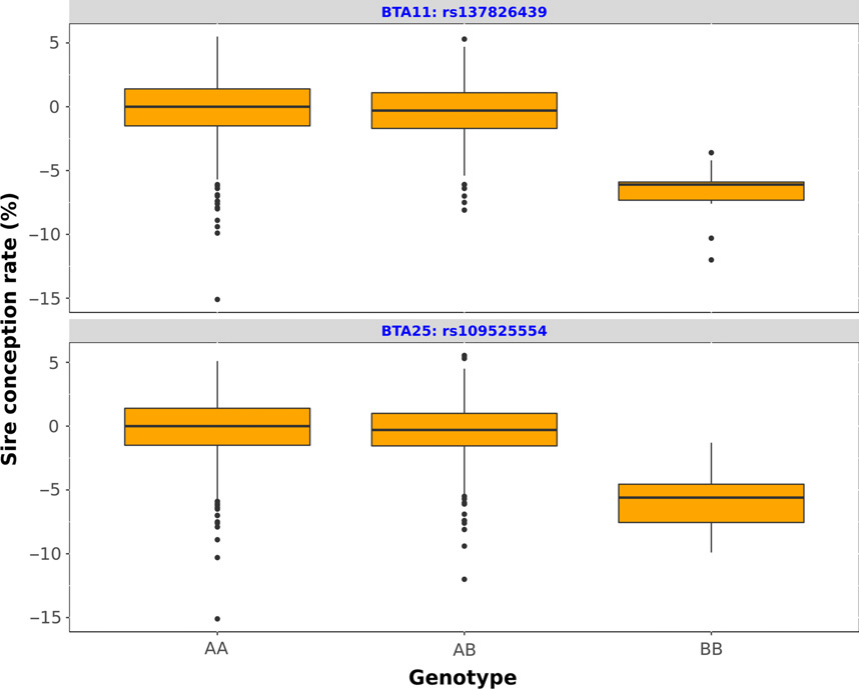
Box plot showing the distribution of sire conception rate phenotypes for two SNP loci with marked recessive effects.

The distribution of SCR values for two SNP loci with marked recessive effects, rs137826439 and rs109525554, is shown in Fig. 3. Notably, these box plots demonstrate that the BB genotypes have much lower SCR values than do genotypes AA and AB. Each of these loci explain differences in conception rates of almost 6%. Unsurprisingly, the BB genotypes are in very low frequency in the population (Table 1), although the B alleles have frequencies between 9% and 14%.

### Gene‐set analysis

A total of 54 763 of the 95 705 examined SNP markers were located within or surrounding 19 792 annotated genes in the UMD 3.1 bovine genome sequence assembly. A subset of 502 of these 19 792 genes was defined as associated with bull fertility.

A panel of KEGG pathways, GO categories and MeSH terms significantly enriched with genes associated with SCR is described in Table 2. Noticeably, some of the KEGG pathways are closely related with sperm physiology, including gap junction (KEGG:04540), calcium signaling pathway (KEGG:04020), MAPK signaling pathway (KEGG:04010) and fatty acid degradation (KEGG:00071). Moreover, several of the significant GO categories are directly involved in sperm biology and fertilization process. For instance, fucosylation (GO:0036065), regulation of cation channel activity (GO:2001257), cilium assembly (GO:0060271), pyrophosphatase activity (GO:0016462), calcium ion binding (GO:0005509) and retinoid binding (GO:0005501) are terms highly associated with spermatogenesis and sperm motility. On the other hand, membrane fusion (GO:0061025), vesicle organization (GO:0061025) and GTPase activity (GO:0003924) are implicated in the acrosome reaction, a crucial process during sperm–oocyte fusion. Finally, a set of MeSH terms were also enriched with genes associated with bull fertility, such as alpha‐mannosidase (D043323), calcium channels (D015220), calcium‐transporting ATPases (D000252) and GTP‐binding protein alpha subunits (D043802), all processes implicated in sperm biology.

**Table 2:** Functional terms significantly enriched with genes associated with sire conception rate is US Jersey Cattle

Term ID	Term	No. genes	Significant genes (*n*)	*P*‐value	Significant genes
KEGG pathways					
04540	Gap junction	73	8	<0.001	*ADRB1, MAP3K2, DRD2, GNA11, PDGFA, TUBB4A, PDGFD, GUCY1A2*
00230	Purine metabolism	137	8	0.024	*PRIM2, PDE4C, ADK, PDE7B, PDE11A, AK7, ENPP3, GUCY1A2*
04020	Calcium signaling pathway	142	8	0.028	*ADRB1, ATP2B, GNA11, SLC8A1, CACNA1A, GNA15, BDKRB2, TNNC1*
04010	MAPK signaling pathway	208	10	0.040	*CACNB4, MAP3K2, MAP3K5, CACNA2D2, PLA2G2F, PDGFA, CACNA1A, MKNK2, FLNB, RASGRF2*
00071	Fatty acid degradation	32	3	0.047	*ACSBG2, ACSL6, CPT1A*
GO: biological processes					
0036065	Fucosylation	6	2	0.009	*FUT8, FUT4*
2001257	Regulation of cation channel activity	20	3	0.013	*EPO, GAL, DRD2*
0006101	Citrate metabolic process	21	3	0.013	*DLAT, SDHAF2, PDHB*
0061025	Membrane fusion	60	5	0.018	*RAB3A, DYSF, DNM2, SNAP29, RAB8A*
0060271	Cilium assembly	64	5	0.023	*POC1A, SNAP29, TMEM216, TMEM138, RAB8A*
0061025	Vesicle organization	72	5	0.036	*INSIG1, RAB3A, DYSF, SNAP29, RAB8A*
GO: molecular function					
0003924	GTPase activity	87	7	0.006	*RAB6B, ARL2, RAB3A, GNA11, DNM2, TUBB4A, RAB8A*
0016462	Pyrophosphatase activity	235	13	0.007	*RAB6B, ARL2, DDX19A, DDX52, RAB3A, ASNA1, GNA11, DNM2, ENPP3, G3BP1, ERCC3, TUBB4A, RAB8A*
0005509	Calcium ion binding	180	10	0.017	*PROC, CAPS, DAG1, DYSF, SLC8A1, SPARC, CALR, ANXA8L1, TUBB4A, TNNC1*
0008083	Growth factor activity	50	4	0.038	*GDF10, IL34, PDGFA, MANF*
0005501	Retinoid binding	13	2	0.042	*RBP3, RBP1*
MeSH: chemicals and drugs					
D043323	Alpha‐mannosidase	4	3	<0.001	*MAN2B1, RAB3A, CALR*
D018832	Molecular chaperones	37	4	0.019	*CRYAB, ARL2, TBCA, CALR*
D015220	Calcium channels	39	4	0.022	*CACNB4, RAB3A, CACNA1A, TRPC3*
D000252	Calcium‐transporting ATPases	11	2	0.035	*SLC8A1, TRPC3*
D043802	GTP‐binding protein alpha subunits	12	2	0.041	*GNA11, GNA15*

## Discussion

Bull fertility has been recognized as an important factor affecting herd fertility in dairy cattle. Previous studies have successfully identified potential genes and pathways affecting service sire fertility in Holstein breed (e.g., Blaschek *et al*. 2011; Han & Peñagaricano 2016; Whiston *et al*. 2017; Nicolini *et al*. 2018). This knowledge, however, cannot be transferred directly to Jerseys because this breed may exhibit differences in the pool of causative mutations, the extent of linkage disequilibrium, the phase between markers and causative mutations and the effects of the causative mutations. As such, the present study was conducted specifically to unravel the genomic architecture underlying SCR, an accurate phenotypic measure of bull fertility, in the US Jersey breed.

The ssGBLUP method revealed 10 genomic regions on seven different chromosomes that explain significant amounts of additive genetic variance. Interestingly, most of these regions harbor genes with functions associated with male fertility. For instance, three putative genes related to Jersey sire fertility, namely *EPB41L2* (BTA9; 69.9–71.4 Mb), *PDGFD* (BTA15; 3.7–5.2 Mb) and *IP6K1* (BTA22; 50.3–51.8 Mb), are directly implicated in testis development and spermatogenesis. *EPB41L2* encodes a membrane protein expressed in Sertoli cells that mediates cell–cell contact between Sertoli cells and germ cells during spermatogenesis (Yang *et al*. 2011). *PDGFD* is a member of the platelet‐derived growth factor family, which plays important roles in the regulation of prenatal and postnatal development of the male gonad (Basciani *et al*. 2010). *IP6K1*, a member of the inositol phosphokinase family, is highly expressed in round spermatids and is essential for histone removal and sperm head elongation during spermatid differentiation (Malla & Bhandari 2017). Moreover, two candidate genes—*PKDREJ* (BTA5; 117.6–119.1 Mb) and *STX2* (BTA17; 45.9–47.4 Mb)—are directly involved in the acrosome reaction, a crucial step during the fertilization. *PKDREJ* encodes a sperm surface receptor that modulates G protein signaling and mediates sperm–egg interaction (Hamm *et al*. 2007). *STX2* encodes a member of the SNARE protein family that controls the membrane fusion during the sperm acrosome reaction (Hutt *et al*. 2005). Finally, two candidate genes—*ZMYND10* and *SLC25A20*—located on BTA22 at 50.3–51.8 Mb, are directly implicated in sperm motility. *ZMYND10* is almost exclusively expressed in the testis, plays a key role in cilia integrity and has been implicated in sperm dysmotility and male infertility (Moore *et al*. 2013). *SLC25A20* encodes a carnitine carrier in the mitochondrial membrane that is involved in ATP production and cell energy metabolism and has been associated with human asthenozoospermia, aka low sperm motility (Asghari *et al*. 2017). Overall, our findings provide the foundation for future fine mapping and functional studies that seek to reveal the specific roles of this set of candidate genes in Jersey bull fertility.

It is believed that non‐additive effects are relevant for fitness‐related traits such as reproduction. Indeed, our recent study revealed significant dominance effects for SCR on Holsteins (Nicolini *et al*. 2018). Here, using an efficient two‐step mixed‐model‐based approach, we identified two genomic regions on BTA11 (2.6 Mb) and BTA25 (19.2 Mb) with significant recessive effects. Notably, these regions harbor genes *FER1L5*,* CNNM4* and *DNAH3,* which have direct roles in male fertility. *FER1L5* encodes a member of the ferlin family that regulates calcium‐mediated membrane fusion events during spermatogenesis (Washington & Ward 2006). *CNNM4* encodes a magnesium transporter that regulates calcium homeostasis during the sperm capacitation and is essential for ensuring sperm fertilizing ability (Yamazaki *et al*. 2016). Mutations in the *DNAH1* gene, which encodes a member of the dynein family, cause multiple abnormalities of the sperm flagella, resulting in impaired sperm motility and, hence, male infertility (Ben Khelifa *et al*. 2014). These findings provide further evidence for the importance of non‐additive effects in fertility traits.

Genomic scans typically detect only major regions, while the vast majority of the genetic variants remain hidden. Therefore, complementary approaches are needed to fully reveal the genetic architecture underlying a complex phenotype. Here, alternative gene‐set analyses were performed to identify biological processes and molecular mechanisms responsible for the SCR variation in US Jersey bulls. Interestingly, gene sets directly related to calcium regulation and calcium signaling were among the most significant functional terms. Calcium is implicated in different sperm physiological responses including sperm maturation, sperm motility and sperm capacitation (Darszon *et al*. 2011). Functional categories closely related to the acrosome reaction, such as membrane fusion and vesicle organization, showed a significant enrichment of genes associated with SCR. The acrosome reaction allows the sperm to penetrate the zona pellucida and fuse with the oocyte membrane (Brucker & Lipford 1995). Many terms related to sperm cell energy metabolism, including fatty acid degradation and pyrophosphatase activity, were also enriched with genes related to SCR. In this sense, recent studies have suggested that lipid metabolism, especially mitochondria fatty acid beta‐oxidation, contributes to ATP production for sperm motility (Amaral *et al*. 2013). Likewise, it is now known that the inorganic pyrophosphate pathway is an important component of sperm physiology, including as alternative energy source (Yi *et al*. 2012). Finally, the MAPK signaling pathway was significantly associated with Jersey bull fertility. Interestingly, it is well‐documented that the MAPK cascades are involved in several male reproductive processes, such as spermatogenesis, sperm maturation, sperm capacitation and the acrosome reaction (Li *et al*. 2009). Overall, our findings provide further evidence that gene‐set analyses can greatly complement whole‐genome scans in understanding biological processes and genetic mechanisms affecting complex traits. As proposed by Abdollahi‐Arpanahi *et al*. (2017), these significant gene‐set terms could be incorporated into genomic prediction models to facilitate the identification of high‐fertility bulls.

It should be noted that none of the ten 1.5‐Mb SNP windows nor the two recessive SNP loci were previously reported as significantly associated with bull fertility in Holstein cattle. This may be due to multiple causes: the major mutations affecting bull fertility in Jersey are not segregating in Holstein, these causative mutations are indeed segregrating in Holstein but are not in high linkage disequilibrium with the SNP markers or simply false‐positive/false‐negative results. However, most of the gene sets significantly associated with SCR in Jersey were also identified as relevant functional terms affecting Holstein bull fertility (Peñagaricano *et al*. 2013; Han & Peñagaricano 2016). Indeed, terms such as calcium ion binding, calcium channels, pyrophosphatase activity and GTPase activity, among others, were significantly enriched with genes associated with SCR in both breeds. These results provide further evidence that biological processes and molecular pathways, rather than single genes, are the primary targets of selection.

## Conclusions

We performed an integrative genomic analysis to dissect the genetic basis of SCR in US Jersey dairy cattle. Ten different regions distributed on BTA1, BTA5, BTA9, BTA11, BTA13, BTA15, BTA17 and BTA22 explained significant amounts of additive genetic variance. In addition, two regions on BTA11 and BTA25 showed marked recessive effects. Interestingly, most of these genomic regions harbor genes that play key roles in male reproduction, including testis development, spermatogenesis, sperm motility and fertilization process. Moreover, the gene‐set analysis revealed functional gene sets, such as calcium signaling, calcium channels, membrane fusion, pyrophosphatase activity, GTPase activity and MAPK signaling, that are directly related to spermatogenesis, sperm cell energy metabolism and acrosome reaction. Overall, this comprehensive study unraveled genetic variants and biological pathways responsible for the variation in Jersey bull fertility. Our findings can provide opportunities for improving service sire fertility in Jersey cattle via marker‐assisted selection.
